# The effects of polio eradication efforts on health systems: a cross-country analysis using the Develop–Distort Dilemma

**DOI:** 10.1093/heapol/czab044

**Published:** 2021-04-21

**Authors:** Daniela C Rodriguez, Abigail H Neel, Yodi Mahendradhata, Wakgari Deressa, Eme Owoaje, Oluwaseun Akinyemi, Malabika Sarker, Eric Mafuta, Shiv D Gupta, Ahmad Shah Salehi, Anika Jain, Olakunle Alonge

**Affiliations:** Department of International Health, Johns Hopkins Bloomberg School of Public Health, 615 N. Wolfe Street, 8_th_ Floor, Baltimore, MD 21205, USA; Department of International Health, Johns Hopkins Bloomberg School of Public Health, 615 N. Wolfe Street, 8_th_ Floor, Baltimore, MD 21205, USA; Center for Tropical Medicine, Faculty of Medicine, Public Health and Nursing, Universitas Gadjah Mada, Sekip Utara, Yogyakarta 55281, Indonesia; Department of Preventive Medicine, School of Public Health, Addis Ababa University, P. O. Box 9086, Addis Ababa, Ethiopia; College of Medicine, University of Ibadan, AddL P.M.B 3017 G.P.O Ibadan, Nigeria; College of Medicine, University of Ibadan, AddL P.M.B 3017 G.P.O Ibadan, Nigeria; BRAC James P Grant School of Public Health, BRAC University, 68 Shahid Tajuddin Ahmed Sharani, Mohakhali, Dhaka-1212, Bangladesh; Heidelberg Institute of Global Health, Heidelberg University, Im Neuenheimer Feld 672, 69120 Heidelberg, Germany; Kinshasa School of Public Health, University of Kinshasa School of Public Health, Kinshasa, The Democratic Republic of Congo; Indian Institute of Health Management Research, 1 Prabhu Dayal Marg, Near Sanganer Airport Terminal 1, Jaipur 302029, India; Global Innovation Consulting Services, Kabul, Afghanistan; Department of International Health, Johns Hopkins Bloomberg School of Public Health, 615 N. Wolfe Street, 8_th_ Floor, Baltimore, MD 21205, USA; Department of International Health, Johns Hopkins Bloomberg School of Public Health, 615 N. Wolfe Street, 8_th_ Floor, Baltimore, MD 21205, USA

**Keywords:** Polio, health systems, political economy, context

## Abstract

Vertical disease control programmes have enormous potential to benefit or weaken health systems, and it is critical to understand how programmes’ design and implementation impact the health systems and communities in which they operate. We use the Develop–Distort Dilemma (DDD) framework to understand how the Global Polio Eradication Initiative (GPEI) distorted or developed local health systems. We include document review and 176 interviews with respondents at the global level and across seven focus countries (Afghanistan, Bangladesh, Democratic Republic of Congo, Ethiopia, India, Indonesia and Nigeria). We use DDD domains, contextual factors and transition planning to analyse interactions between the broader context, local health systems and the GPEI to identify changes. Our analysis confirms earlier research including improved health worker, laboratory and surveillance capacity, monitoring and accountability, and efforts to reach vulnerable populations, whereas distortions include shifting attention from routine health services and distorting local payment and incentives structures. New findings highlight how global-level governance structures evolved and affected national actors; issues of country ownership, including for data systems, where the polio programme is not indigenously financed; how expectations of success have affected implementation at programme and community level; and unresolved tensions around transition planning. The decoupling of polio eradication from routine immunization, in particular, plays an outsize role in these issues as it removed attention from system strengthening. In addition to drawing lessons from the GPEI experience for other efforts, we also reflect on the use of the DDD framework for assessing programmes and their system-level impacts. Future eradication efforts should be approached carefully, and new initiatives of any kind should leverage the existing health system while considering equity, inclusion and transition from the start.

**Key messages**

- The Global Polio Eradication Initiative (GPEI) triggered developments, distortions and mixed effects in local health systems, reflecting the adaptive nature of a programme that developed capacity and systems where they were lacking but also distorted incentives for individuals and organizations in its mission to reach eradication, in particular when faced with weaker routine immunization and health systems.
- Despite fears that the polio programme’s achievements cannot be sustained without transition planning, there are persistent concerns at the global level that transition planning will draw attention away from eradication efforts leading to inconsistent and delayed efforts on transition.
- Future disease control programmes need to carefully consider the role of global actors driving the agenda, the need for transition planning, integrating into local programmes rather than supplanting them, and sustaining key commitments to equity and inclusion.
- The Develop–Distort Dilemma (DDD) framework was a useful analytical tool for evaluating the GPEI’s interaction with health systems, in particular for identifying unintended consequences; we recommend adding domains for integration and parallelism and incorporating the DDD with other systems thinking tools.

## Introduction

Established in 1988, the Global Polio Eradication Initiative (GPEI) set an ambitious goal of eradicating poliovirus globally by the year 2000 (World Health Assembly, 1988) utilizing a strategy of (1) routine polio immunization, (2) supplementary immunization activities (SIAs), (3) mop-up campaigns targeting high-risk areas and (4) surveillance. World Health Assembly member states and the GPEI partner consortium [World Health Organization (WHO), United Nations Children’ Fund (UNICEF), U.S. Centers for Disease Control (CDC), Rotary International and eventually the Bill & Melinda Gates Foundation], have successfully reduced the global polio burden by more than 99% (CDC, 2020). Still, a growing number of countries are experiencing outbreaks of circulating vaccine-derived poliovirus (cVDPV), and repeated missed deadlines have frustrated countries and implementers (The Lancet Infectious, 2013).

In the 1990s, critical questions were raised about the initiative’s commitment to strengthening routine systems including whether developing countries should allocate resources to polio if it was a low-priority issue locally, whether donors would contravene their own historical patterns and truly invest in sustainable development and—presciently—as polio cases dwindled, whether the focus would shift to strictly promoting eradication activities (Taylor *et al.*, 1997). These drew on the understanding that vertical programmes like the GPEI tend to have short time horizons and external funding, and circumvent system constraints rather than addressing them (Oliveira‐Cruz *et al.*, 2003), but still may be desirable as a stop-gap measure when quick action is needed or to address the needs of hard-to-reach and vulnerable populations (Atun *et al.*, 2008). The response from polio eradication advocates focused on the programme’s successes, stating that system strengthening was ‘a desirable secondary gain’ of eradication investments (Lee *et al.*, 1998) and noting that both system strengthening and polio activities needed adequate funding (Sutter and Cochi, 1997). By the early 2000s, polio efforts split from the Expanded Programme on Immunization (EPI) at the global level. Today, the Polio Endgame Strategy (2019–2023) remains focused on the GPEI’s eradication aim, addressing risks to success (insecurity and conflict, weak and fragile health systems, and operational and management issues), while also increasingly considering issues related to integration with routine immunization (RI), asset transition, containment and certification (WHO, 2019).

Over the years, researchers have evaluated the impact of polio eradication activities on specific health system ‘hardware’, e.g. health information systems like the global laboratory network (Macaulay and Verma, 1999) and immunization financing (Levin *et al.*, 2002), and assessed the impacts on various aspects of health service delivery, such as effects on primary health care (Closser *et al.*, 2014), management capacity, partnerships and collaboration, social mobilization (Loevinsohn *et al.*, 2002; Arora *et al.*, 1999; Mogedal and Stenson, 1999), and surveillance and outbreak response (Vaz *et al.*, 2016; Kandel *et al.*, 2016). Social and political determinants that play a role in marginalization (e.g. gender, household income, religious and cultural beliefs, power relations and trust) have also been shown to affect implementation of immunization programmes, including polio (Glatman-Freedman and Nichols, 2012; Taylor, 2009).

These studies have demonstrated positive effects of the polio eradication initiative on health systems; they have also found that better planning could have mitigated disruptions to the health system and that opportunity costs—caused by either a distraction from other health priorities or a lack of deliberate effort to institutionalize investments into the health system—have abounded. Increasingly, researchers have come to understand the ways in which context and health systems ‘software’ [(i.e. the interests, values and norms, and power dynamics that underpin system function (Sheikh *et al.*, 2011)] have both stymied polio eradication and mediated the initiative’s effects on health systems (Mshelia *et al.*, 2020). Further, by engaging in a ‘culture of optimism’ where evidence of achievability is overvalued, the GPEI has perpetuated framing polio eradication as always imminently at hand while avoiding critical implementation challenges (Closser, 2012). White has framed this problematic rhetoric in endgame campaigns as the language of concentrated effort that focuses on needing ‘just a few more years’ to reach its goals, when it is neither realistic nor appropriate (White, 2020).

Global actors have also increasingly acknowledged that fragmentation, an ‘enduring feature of the global health landscape’ over the last several decades, may undermine the drive toward universal health coverage (Ooms *et al.*, 2018). While numerous methodologies have been deployed to try and understand discrete effects of programmes like the GPEI on health systems, there is a need to deploy health systems tools that can systematically describe the impacts of verticalization and point to ways to ‘undo’ some of the fragmentation that currently exists in the global health landscape.

Our analysis focuses where narrow programme objectives are often elevated above system-level concerns. This phenomenon, referred to as the ‘Develop–Distort Dilemma’ (DDD), suggests that well-intentioned health interventions may have deleterious effects on health systems when interventions fail to account for the full context in which they are implemented and when the ‘immediacy of interest’ of health targets supersedes long-term efforts to strengthen health systems (Peters *et al.*, 2009; Subramanian *et al.*, 2011). The DDD framework has been used to describe health market failures and to improve planning processes (Bloom *et al.*, 2011; Hitchins *et al.*, 2004; Peters *et al.*, 2012). It has also been proposed as potentially useful for programmatic assessment of the impact of large global programmes on health systems. In this paper, we offer the first such assessment as we draw on country- and global-level evidence to understand the impact of the polio eradication efforts on health systems through a specific lens of developments and distortions in order to inform future programme design and implementation.

This analysis is part of a larger implementation science study, ‘Synthesis and Translation of Research and Innovations from Polio Eradication (STRIPE)’, documenting lessons learned from polio eradication efforts at the global level and in seven study countries (Afghanistan, Bangladesh, DRC, Ethiopia, India, Indonesia and Nigeria) (Alonge *et al.*, 2020). The seven countries included in this analysis represent various GPEI context typologies (e.g. endemic, outbreak, at-risk and polio-free), as well as considerable variability in size, population density, income status and health systems (Table 1). While other components of the STRIPE study examine implementation barriers and strategies, this analysis focuses on the interaction between the polio eradication initiative and health systems.

**Table 1.** Demographic, health systems and polio eradication programme characteristics, by country

Indicator^a^	Afghanistan	Bangladesh	Democratic Republic of Congo	Ethiopia	India	Indonesia	Nigeria
Population, millions (2018)	37.1	161.3	80.0	109.2	1003	267.6	195.8
GNI per capita (2015)^b^	600	1220	460	600	1600	3430	2880
Under-5 mortality rate per 1000 live births (2015)	73.1	36.4	51.3	61.2	44.1	27.2	107.5
Maternal mortality ratio per 100 000 live births (2015)	396	176	442	353	174	126	814
Fragility index (2015)^c^	107.9	91.8	109.7	97.5	79.3	75.0	102.5
Health systems indicators							
Current health expenditure as % of GDP (2015)	10.3	3.5	7.81	4.73 (2013/14)	4.69	3.35	3.56
OOP payments as % of current health expenditure (2015)	78.4 (2011)	61 (2011)	38	33 (2013/14)	41	48.3	72.24
Number of hospital beds per 10 000 population^d^	5 (2015)	8 (2015)	8 (2006)	3 (2015)	7 (2011)	12 (2015)	5 (2004)
Number of clinical health workers (doctors, nurses, midwives) per 10 000 population^d^	6.2 (2014)	7.4 (2015)	5.6 (2013)	9.4 (2017)	28.9 (2017)	24.4 (2017)	18.4 (2013)
Polio eradication programme							
Polio country status^e^ at initiation of data collection (2018)	Endemic	Polio-free (2014)	Outbreak	At-risk	Polio-free (2014)	Polio-free (2014)	Endemic
Polio country status at time of publication (2020)	Endemic	Polio-free (2014)	Outbreak	Outbreak	Polio-free (2014)	At-risk	Outbreak
Oral poliovirus third dose (OPV3) coverage 2000–2018 (%)^f^	24–73	83–98	42–79	55–67	57–89	72–80	31–57
Inactivated polio vaccine first dose (IPV1) coverage (2018) (%)^f^	66	75	79	52	75	66	57
External support	Extensive; government only covers 5% of immunization programme	GPEI ending in 2019 Gavi ending in 2022	Substantial financing from external sources	Substantial financing from external sources, mainly GPEI which is ramping down. Resource mobilization includes engaging other donors to support programme	External support from Gavi, GPEI and others but most from government of India	Funded by government of Indonesia and Gavi support (2004–2017)	External support from WHO, UNICEF, Rotary, CDC and Gavi

a All data from country programme summaries and HiT tools unless otherwise noted.

b Data from World Bank Data: https://data.worldbank.org/country.

c Fragile States Index: https://fragilestatesindex.org/country-data/.

d Data from WHO Global Health Observatory: http://apps.who.int/gho/data/node.main.

e A number of polio status changes occurred between the initiation of data collection and publication. At the initiation of research, Ethiopia was classified as ‘at-risk’ but subsequently confirmed two cases of circulating vaccine-derived poliovirus (cVDPV) in May 2019, linked to an ongoing outbreak in Somalia. Indonesia also confirmed one case of cVDPV in Papua province in November 2018 and was temporarily re-classified as an outbreak country; Indonesia was deemed as no longer infected in June 2020 and re-classified as an at-risk country vulnerable to reinfection. Finally, in August 2020, Nigeria was declared wild poliovirus (WPV)-free along with the entire WHO AFRO region after 4 years without a WPV case.

f WHO/UNICEF Estimates of National Immunization Coverage (WUENIC).

## Materials and methods

### Develop–Distort Dilemma framework

**Figure 1.**
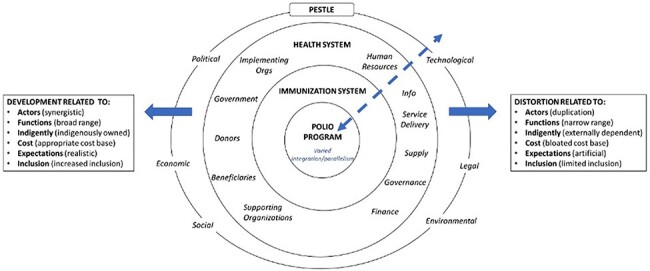
Develop–Distort Dilemma framework adapted for assessing the polio eradication programme (adapted from Peters *et al.,* 2012)

Figure 1 represents our adaptation of the DDD framework. Our goal was to evaluate changes to the health system precipitated by polio eradication activities according to the existing DDD framework, in part to assess the utility of the DDD as a programme assessment tool for systematically describing the impact of large global programmes on health systems. As such, we retained the categories of potential developments and distortions of the health market system summarized by Peters *et al.* as the basis of our analysis (Peters *et al.*, 2012) (Table 2). Within each of these ‘domains’, an intervention may have beneficial effects (developments), detrimental effects (distortions) or both (mixed effects) on the health system. Within the domain of inclusion, e.g., an intervention into the system may expand (develop) or limit (distort) the accessibility of health services to the population.

**Table 2.** Develop–Distort Dilemma domains

Domain	Definition
Actors	Stakeholders across public and private sectors (for and not-for-profit) who are involved in and/or influence financing, design or implementation of health activities within the polio programme or the broader health system
Functions	Scope of the activities that enable delivery of the programme
Expectations	Expectations about what the programme’s activities could include and achieve, including responsibility for different components
Inclusion	Degree to which the programme actively ensures that all potential beneficiary groups receive programme services
Indigeneity	Degree to which the programme’s activities are owned/resourced by indigenous country actors relative to external actors
Cost	How much the programme and its associated activities cost

The DDD framework’s original focus on market systems required adaptation since the supply and demand dynamics are different for a public or semi-public good (e.g. a free, worldwide immunization programme) than for private goods (e.g. contraceptives). In addition, which actors are relevant, how they interact, and their relative power is specific to the intervention and system of interest, and reflects multi-layered system dynamics. For our purposes, it was most important to capture the interactions between multiple organizational layers (the polio eradication programme, immunization and health system, and the broader context) through which developments and distortions may have occurred. We retained the core exchange of health goods or services at the centre of the system (i.e. polio vaccination of the beneficiary) but expanded the description of the health system to include the most relevant supporting functions, stakeholders and contextual factors. While these are represented as overlapping layers in Figure 1, we recognize that programme activities and the implementing environment influence each other in a bidirectional relationship and that the level of embeddedness of these layers (e.g. degree of integration or parallelism of the polio programme within the health system) may vary in practice.

### Data sources

This paper represents the results from a cross-case analysis of seven country case studies and global data, which included documentary data and key informant interviews (KIIs). Country teams developed a programme summary from the document review that captured polio programme dimensions and an adapted Health systems in Transition (HiT) tool to capture health system performance indicators at different time points (WHO—Regional Office of the Western Pacific, 2016).

Key informants were selected to ensure representativeness of key bodies (e.g. national and subnational government entities, implementing organizations, donors and frontline health workers) and areas of expertise (e.g. advocacy and communications, and surveillance), and were drawn from a previously enumerated sample of individuals involved in polio eradication activities between 1988 and 2019 (Peters *et al.*, 2020). Interviews were conducted in English or the local language with subsequent translation. Table 3 presents the full sample of respondents.

**Table 3.** Interview respondent characteristics, total sample

	Country		Global		Total	
	*n*	%	*n*	%	*n*	%
Data						
No. of interviews completed	177	–	18	–	194	–
No. of interviews included in analysis	177	–	17^a^	94.4	193	99.5
Level						
Global actors	0	0	17	100.0	17	8.8
National actors	84	47.5	0	0	84	43.3
Subnational actors	71	40.1	0	0	71	36.6
Frontline health workers	22	12.4	0	0	22	11.3
Affiliation						
Academic organization	4	2.3	1	5.9	5	2.5
Consulting firm	1	0.6	0	0	1	0.5
Government	96	54.2	0	0	96	49.5
GPEI partners	45	25.4	14	82.3	59	30.4
Implementing organization	21	11.9	2	11.8	23	11.9
Other (e.g. clinical)	10	5.6	0	0	10	5.2
Gender						
Female	46	26.0	4	23.5	50	25.8
Male	131	74.0	13	76.5	144	74.2


a One respondent’s interview was inaudible and could not be reviewed for analysis.

### Analysis

A codebook to guide deductive analysis of the KIIs was developed drawing on (1) the six DDD framework domains; (2) WHO’s health systems building blocks (WHO, 2007); (3) transition planning that covered asset integration (e.g. surveillance, laboratories, cold chain infrastructure, human resources and communications capacity), institutionalization of polio programme efforts (i.e. extent to which practices and innovations are reflected in system norms, standards and practices) and broader challenges to transition (e.g. active or potential threats related to surveillance, stockpiling, outbreak management and financing) (Bao *et al.*, 2015) and (4) broader contextual factors. Codes capturing the directionality of any changes reported (e.g. effect from polio and effect on polio) and non-binary impacts (e.g. ‘mixed effects’, where the consequence is not exclusively a development or a distortion) were included in the original codebook as well. Finally, in addition to the existing DDD domains, we added codes for ‘integration’ and ‘parallelism’ to capture the impact of the eradication programme’s delivery strategy, i.e. its level of embeddedness within existing structures.

Coding was conducted by four analysts of the central STRIPE team using the electronic software program Dedoose (version 8.2.27). The codebook was piloted, refined and clarified by three coders to ensure inter-coder agreement. In addition to audio recordings and transcripts, case summaries describing key themes were developed to ensure an audit trail and were discussed to identify points of convergence and divergence within and across cases. Since none of the coders have participated in GPEI implementation or represented the study countries, findings were further validated with country teams and the Technical Advisory Committee of the parent study to ensure accurate interpretation of respondents’ views. The parent study was determined to be non-human subjects’ research by the Johns Hopkins School of Public Health Institutional Review Board and was reviewed and approved by local ethics boards in each country.

## Results

The results are organized according to the six domains of development and distortion, as well as transition planning. Results presented here focus on findings that apply to multiple countries, even if manifested in different ways, or that apply to one or two countries but play an outsize role in the relationship between the polio programme and the health system. KIIs generated no data on programme cost, so this is not explored. Integration and parallelism cut across each of the existing DDD domains, so we have incorporated these findings throughout.

### Development and distortion

#### Actors

We found both development and distortion relating to actors, with varied impacts at different levels of the system. At the global level, the GPEI became more democratic in its decision-making beginning in 2012 as it moved away from a model where operational decisions were centralized at the WHO. While this shift to shared decision-making increased the management burden, partners perceived the evolution as worthwhile because it led to a clearer division of roles aligned with each partner’s strengths and allowed for more accountability (i.e. checks and balance) between GPEI partners.

*There is probably an opportunity cost associated with the partnership and the amount of conference calls and meetings…we need to have, which possibly sort of affects our ability to move faster. But…weighing the pros and cons, we still feel that this is the way it needs to work…it’s not as easy as having a dictator that decides everything and knows everything and the others follow* (GlobalDR-03).

Most recently, the introduction of IPV resulted in the addition of Gavi to the GPEI to help link polio to RI; while polio eradication and RI have quite different mandates, Gavi is intended to play a significant role in facilitating the integration of polio eradication activities and RI. How its inclusion will affect the shared decision-making within the partnership remains to be seen.

At a national level, learning and policy transfer between countries was also seen as a key development in India and Indonesia, where respondents explicitly discussed learning from other countries’ polio eradication experiences through conferences, stakeholder meetings and country and site visits.

Synergy among global partners did not always translate to development at the national and sub-national levels. The initial WHA polio declaration did not have widespread buy-in among target countries—often seen as ‘partners’ work’ because of misalignment with national priorities. This resulted in uneven country ownership on eradication, especially as polio efforts diverted attention and resources from other local priorities. Further, overlapping functions between GPEI partners at the national level persisted and caused tension (e.g. India).

Respondents explained the global governance structure was ‘felt’ in country, and the ground presence of partners was much larger for the polio programme. In some cases, global governance structures ended up supplanting national ones (e.g. Afghanistan and Nigeria) at certain periods when the pressure was ramped up to address implementation issues within those countries. At the sub-national level, polio campaigns consistently diverted staff from their usual tasks, causing the *de facto* de-prioritization of RI and primary health services. Monetary incentives for polio campaign workers exacerbated this distortion such as in the DRC where health workers implementing polio campaigns received higher compensation than those delivering other health campaigns. This resulted in draining workers from the health system and dwarfing attention to other health programmes, especially those that were primarily government funded.

#### Functions

We found instances of both development and distortion of key health system functions. The polio programme contributed to the expansion of health system functions in several ways. First, capacity building for polio activities contributed to other health programmes when polio-trained staff have moved elsewhere in the system, taking their skills with them, including for emergency response. For example, the Ebola outbreak in Nigeria in 2014 was quickly curtailed using experienced polio staff to do contact tracing and other field-level operations that minimized disease transmission, while community health workers have been tapped to assist with cholera- and malaria-related service delivery.

Second, respondents described how the polio programme built accountability mechanisms and a ‘data-driven’ focus that could reach down to the local level, especially around health systems management personnel. Improvements to system functions were most notable where the influence on other programmes was clearly felt.

*Polio program has helped to build [the] surveillance system, it helped to build laboratory network capacity. See now the same laboratories are working on measles, they are working on rubella, they are working on other infectious disease because they found it so valuable to have [a] laboratory close to field so they can test and confirm cases* (IndonesiaLA12).

This impact occurred most often where the health system (or other health programmes) was able to piggyback on polio eradication efforts to support other functions, including using surveillance systems for other vaccine-preventable diseases (e.g. Bangladesh transforming the national polio lab for measles and rubella surveillance), integrating delivery and cold chain functions in Ethiopia or using polio programme structures to deliver other programmes (e.g. India for nutrition and DRC for malaria net distribution, vitamin A, etc.) In Colombia, a cholera outbreak in 1990 prompted a shift from polio immunization day to Hygiene Day, with expanded focus on water and sanitation, suggesting increasing ownership over the programme (see also Indigeneity section).

Despite these instances of ‘accidental’ or organic integration, the polio programme increasingly operated in parallel to RI services, leading to a narrowing of functions within the health system, even as they were expanded within the polio programme infrastructure. Importantly, the extent and nature of this distortion depended on the relative strength of the system at the start of eradication efforts. ‘Strong’ health systems integrated polio into the system more fully, while in ‘weak’ health systems, the polio programme essentially became the system’s delivery structure.

Per respondents, the GPEI underestimated how weak health systems would impact programme implementation (especially since Pan American Health Organization region experiences where health systems were relatively strong formed the basis of the GPEI), and by the early 2000s, internal divisions arose regarding the programme’s direction and how narrow or broad its focus should be. Respondents indicated it seemed easier at the time to reach eradication as a separate programme, relying on mass campaigns to reach children that were not otherwise served by the health system:

*It was quite difficult, especially when we had ongoing transmission, to get the program to think other than kind of a zero sum game mentality…In the new strategy, the language is incorporated…to ensure…this mind shift that routine immunization is as important to the sustained poliovirus cessation as SIA’s…**the difficulties in trying…to understand that a day spent on strengthening routine immunization isn’t a day lost <laughs*** ***>*** [GlobalAK07 (emphasis added)].

Parallelism did enable programme success and made it easier to demonstrate progress towards eradication, but de-integrating the programme from EPI at a global level significantly impacted service delivery functions at the national and sub-national levels, especially where the programme operated either partially (India and Ethiopia) or extensively (Afghanistan, DRC and Nigeria) in parallel to the health system.

*The problem is…they separated EPI from polio…For polio eradication the best program is routine strengthening, but it is completely ignored. Our staff prefer to work on polio because we have many facilities in polio: from financial, economics point of view or motivations, it is all in polio…even our officials go to provinces, they tell the staff, ‘you should focus on campaign because it’s an emergency.’ They ignore all about routine* (AfghanistanNAD01).

At the time, separating polio eradication activities seemed expedient as RI would ‘take a generation’, but today lack of integration in some countries (and in some districts) has made it highly challenging to ensure functions developed by the polio programme are sustained within the health system over time. As polio epidemiology and the vaccination strategy have changed, new issues have arisen in areas with poor RI, including how to address cVDPV when IPV coverage is persistently low.

*We have a cohort of children that are being born, almost 8 million every year, maybe 33% go and get the IPV. 67% that don’t go and so their immunity for type 2 is low and so…once there is a cVDPV, they are susceptible…Then people in the inaccessible areas are not even exposed to it, because it’s easier to give the oral one* (NigeriaEO20).

#### Expectations

Regarding the domain of expectations, we found evidence of both distortions and mixed effects. In terms of distortion of local health systems, the main issue has been the pressure generated by the expectation of eradication. The performance expectation of polio efforts (i.e. ‘seriousness’) was different than for RI or other health programmes, with more structured accountability mechanisms. As a result, polio garnered disproportionately more attention from workers.

*…polio was supposed to enhance [the national primary health care development agency], but it has ended up making it difficult for [it] to function because all the attention was on polio. Everybody says we must eradicate polio…we missed the target…twice, there was 2000, we missed the target…they set 2005, we missed it again, so there was a lot of pressure on everybody* (NigeriaAA27).

At the global level, WHO’s and UNICEF’s efforts are seen as ‘too big to fail’, so it has become difficult for them to divest responsibilities, particularly because not all governments have been in a position to take over GPEI-led functions.

Another emerging distortion is linked to how the GPEI has changed its approach to contracting workers intended to support local systems. Global respondents discussed how the WHO shifted to short-term contracts when it felt that eradication was in sight in order to reduce the liabilities on their payroll; however, missed deadlines resulted in temporary, 1-year contracts renewed recurrently for over 10 years. This lack of security has affected morale and, when eradication is achieved, will have significant impacts on country-level health workforce where these contracts are concentrated.

At a community level, the household focus of polio eradication generated expectations that other health services should also be offered door to door, reflecting chronic underfunding of health services. These expectations resulted in mixed effects on the health system depending on the country. In some areas, these could not be met by government and contributed to the erosion of community trust (e.g. Afghanistan and Nigeria); in others, health programmes began piggybacking on polio’s service delivery capacity to offer additional services like vitamin A supplementation or water and sanitation education, which improved access to and demand for health services (e.g. Bangladesh, India and Ethiopia).

#### Inclusion

While the programme hoped to achieve eradication with widespread coverage (e.g. 90% and 99%), missed targets have forced a renewed focus on reaching the hard-to-reach (the last 1%), giving the programme an explicit focus on inclusion and leading to both developments and mixed effects. A strong commitment to reach every child has demonstrated the efforts necessary to achieve inclusion targets in spite of challenges of political and geographic access (e.g. internal conflict, territorial size, underdeveloped transport networks and seasonal flooding). New strategies for reaching inaccessible areas included negotiating vaccination cease fires, setting up immunization booths at transit hubs and cross-border areas, and using satellite mapping to identify remote and nomadic communities.

In Bangladesh, service delivery to vulnerable populations increased because polio made a larger effort than RI to reach every household; likewise, in other settings, health benefits were extended to vulnerable populations via co-delivery. Respondents also noted the polio programme has been able to highlight areas and communities that consistently struggle to receive government services.

***One thing in the polio program, we reached the most marginalized families who were not part of any…health system, they were not in the list of any beneficiaries…survey or anything.*** *They were staying in the outskirts of the village for a few days and then they will move in 15 days, 3 months they will move…So polio program brought each and every group under the umbrella of service delivery for polio organization and now we are delivering…immunization services* [IndiaPN05 (emphasis added)].

It is unclear whether the polio programme would have engaged in these extraordinary efforts if eradication could have been reached with a lower coverage target.

A mixed effect of inclusion has been that efforts did not consistently translate to RI services but instead relied on campaigns to reach vulnerable populations with the polio vaccine.

*Certainly, there’s been a split between the focused effort on polio, mostly delivered through campaigns, and the lack of ability of the immunization program to…increase access in a routine manner to the population in the country. And this is not, I would not say it’s the fault of the polio program, but certainly the program has not helped address this gap* (GlobalDR03).

Respondents across contexts described environmental factors related to topography, population movement and infrastructure, which made it challenging to reach certain communities. Polio was exceptional in its effort to address these barriers—‘compared to the routine vaccination, there are solutions that [have] been made’ (DRCMB19), but extending these successful house-to-house delivery strategies to other health services has proven difficult given the resource intensity required.

#### Indigeneity

The degree of country ownership of the polio programme has varied widely between contexts, impacting health systems in both positive and negative ways. Further, maintaining country ownership and attention after missed goals and past deadlines was difficult.

Polio implementation has shifted over time to be more inclusive of local staff and include communities in decision-making, even when core technical assistance roles are filled by GPEI partners’ contract workers. Further, in countries where polio activities were initially spearheaded by external support, there has been movement towards country-owned polio efforts (e.g. domestic vaccine production in India and Indonesia, establishing governing bodies within Ministries of Health). In some cases, like Indonesia, this movement was a reflection of a broader push to nationalize health programmes rather than a direct result of the polio programme.

Still, most remaining programmes are not indigenously financed or supported. This is partly a function of strategic allocation as country governments direct their own funds to other health priorities. At the same time, some countries are dependent on polio resources to support core functions of the health system (e.g. Afghanistan, Nigeria and DRC). Global-level actors warn that the longer the programme persists, the fewer incentives countries have to take more ownership leaving partners to deal with ‘partners’ priorities’.

*In the instance of GPEI, the money is not going to the government. It’s going to WHO and UNICEF and they are doing jobs that the government should have been doing but they’re basically dis-incentivizing the government from doing them…we talk about WHO and UNICEF as implementing partners, but they are substituting for government services in the public health system and to get out of that will be very difficult* (GlobalDR08).

*All of the money that polio is putting into some of the worst-off countries that have the poorest health systems in order to try to stop polio virus transmission has allowed countries and systems…off the hook in a sense* (GlobalAK07).

From the country’s perspective, implementers indicated that taking over partner-supported functions may not be feasible for their governments in the short term to mid-term and feared the deterioration of services with partner withdrawal (see also Transition section). Across levels, there was little clarity on the actions required to increase country ownership and mitigate distortions caused by external dependency, but it was clear that integration of polio-supported functions will take time and effort. For example, domestic resource mobilization will be a persistent problem both in countries with substantial donor support for health (e.g. Yemen and South Sudan) and in countries where funds might be available but are not allocated to health (e.g. Chad and Angola).

*We worked together with a group of parliamentarians called [redacted for confidentiality] in support of vaccination so that there is a budget line in the state budget for the purchase of traditional vaccines and support vaccination campaigns. Done that way we can already arrive at purchasing traditional vaccines and in provincial governments, we conduct advocacy for provincial governments to ensure still the transportation of vaccines to vaccination sites. So, there are a number of initiatives like this that are developed to build on the achievements of polio control in routine immunization activities* (DRC19).

There is also a persistent question about data ownership between global-level partners and country systems that has not been resolved. Despite GPEI’s intentions for governments to own programme data, respondents report that local data are owned by WHO and not all governments have had capacity to house or analyse the data, partly because systems were established in parallel. In practice, ownership of data systems leads to tension on how problems are framed. Respondents noted that future partnership efforts need to invest in data sharing agreements up front.

### Transition

In terms of transition and legacy planning, especially for countries where polio eradication was well integrated into RI from the outset, such as in the Americas, Indonesia and Bangladesh, post-elimination transition was not a significant shift but rather a continuation of existing RI activities. However, transition planning raises core questions about what is being transitioned, to whom and for how long it should operate. One global-level respondent categorized polio assets into three areas: (1) assets that can disappear, (2) assets that should be transitioned to RI or primary health care services (e.g. outbreak response) and (3) assets that are still required post/near to eradication (e.g. cVDPV monitoring) (GlobalDR04). There was no consensus among respondents about whether the focus should be on activities or staff, how assets should be assigned into each category, how these categorizations should vary between countries and whether countries would be expected to independently monitor and respond to cVDPV outbreaks. Although some aspects of the programme have been institutionalized (e.g. social mobilization maps in India, GIS maps in Nigeria, and laboratory and surveillance networks), the lack of clarity on transition at the global level undermines the overall messaging at country level about polio support eventually coming to an end.

Global-level respondents were inconsistent about whether transition planning should be prioritized, reflecting current tensions between those who feel transition planning should have started years ago and those who want to postpone discussions until eradication is achieved. A Transition Independent Monitoring Board (TIMB) was established in 2017, but respondents indicated that it had met infrequently, in part due to lack of support from key GPEI partners. The TIMB’s last report from December 2018 also states that the Transition Management Group was abolished in July 2018, with WHO assuming leadership for transition and GPEI no longer playing a significant leadership role. Global respondents also questioned whether GPEI partners themselves have been making institutional post-transition plans for themselves.

There was broad-based concern among respondents that polio eradication achievements (e.g. new health workers and changes to performance management) will not be sustained without external support, which is supported by the TIMB’s last report (Polio Transition Independent Monitoring Board, 2018). This concern was acute among country-level respondents who recognized their systems’ dependency on polio financing and technical assistance. Specific areas of concern around legacy planning included integration and sustainability of IPV delivery, government and GPEI health workers moving to other programmes before epidemic control is reached, institutionalization of surveillance and mobilization of sustained financing for post-control efforts.

## Discussion

We found the DDD framework useful for evaluating the GPEI’s interaction with health systems, in particular intended and unintended consequences that should be considered for future global health initiatives. Emerging from our analysis were a number of themes that have been captured elsewhere (Taylor *et al.*, 1997; Vaz *et al.*, 2016; Kandel *et al.*, 2016), including developments related to health worker capacity at multiple levels, improving local performance and accountability systems, and better mapping and enumeration of hard-to-reach communities. Likewise, we found distortions across country settings, such as pulling other health staff into polio campaigns, distorting payment structures for health workers, fostering unachievable community expectations and incentivizing better performance for polio efforts than routine services.

However, the DDD-based analysis also identified new findings such as changes in global governance of the consortium and how these played out at the national level, issues around indigeneity and country ownership of polio activities and their implications for sustainability. The GPEI Governance Review also highlights similar issues including the need for greater accountability and transparency within GPEI and greater country ownership and engagement (GPEI, 2020b). We also found a range of mixed effects that became especially evident as GPEI’s implementation extended over a longer period of time than expected. These mixed effects expose the feedback loops between polio-specific activities and the health system to provide an understanding of how polio programme activities can reinforce and/or counterbalance negative health systems’ impact at the same time. For instance, incentivizing health workers for polio activities may improve polio programme performance but lead to unsustainable expectations for health worker salaries and distort the local payment structure. With the knowledge that vertical programmes are not going away anytime soon, such understanding reveals relevant intervention levers for making vertical programmes work more effectively for health system strengthening.

Our analysis also identifies critical issues related to the sustainability and transition of polio eradication activities, which are not well explored in the literature. The development theory at the centre of the DDD is that external actors play an inherently temporary role to facilitate system-level changes (Centre, 2014), priming the system to maintain key functions and effectively respond to emerging issues over time. We found a lack of consensus on transition planning, at the global and national levels. For countries where polio was integrated with RI from the outset, institutionalization of polio activities was straightforward. For countries still receiving polio eradication support, there is an open question about which activities to institutionalize and how. Worryingly, despite concerns at both global and country levels that some of the gains achieved through the polio programme cannot be sustained without polio programme financing, we did not find a consistent position on when to start transition planning or how much effort to direct towards it while eradication efforts are still ongoing, reflecting internal conflicts on this issue (perhaps the ‘culture of optimism’ and language of concentrated effort are still holding sway). This is further evidenced by the dissolution of the GPEI’s transition management group and subsequent fragmentation of transition efforts, even as the GPEI has placed an increased emphasis on integration in its endgame strategy.^1^ Importantly, several countries are facing multiple donor transitions simultaneously that require better coordination across development partners. The lack of consensus and emphasis on transition planning within the GPEI sets the stage for countries and local polio programmes to be critically unprepared.

Finally, applying the DDD across country cases highlighted how health system dynamics changed over time and varied between contexts. The success of the four-pronged polio eradication strategy in the Americas (i.e. RI, SIAs, mop-up campaigns and surveillance) led policymakers to believe this approach would be transferable to other contexts. In many ways, this has borne out, but the GPEI’s intention to build and support RI systems and utilize SIAs as just that—a supplement—has eroded over time with supplementary campaigns now becoming the main strategy of the programme, especially as it has ramped up efforts to reach the remaining hard-to-reach populations. In strong health systems, external investment caused an amplifying effect: the influx of resources increased the rate of health system development. Over time, this created a positive or reinforcing feedback loop in which programmatic investments led to increased system capacity and eventually improved programmatic outcomes (Paina and Peters, 2012; Rickles *et al.*, 2007). Our study suggests this phenomenon occurred where polio activities were substantially integrated into RI from the outset. Where this occurred, global and local governance worked in tandem to drive the polio programme, and as a result, local governments were better able to sustain core immunization and surveillance functions.

In contexts of weak health systems, however, the balance of activities has skewed away from RI and towards campaigns delivered through parallel systems. While this is a reaction to implementation challenges faced in the field, it is also a product of eradication itself—the desire to achieve eradication drove implementers to work around the system where necessary just as Taylor *et al*. predicted (Taylor *et al.*, 1997). Thus, the separation from RI was driven by contextual factors (e.g. weak health systems) and exacerbated them (e.g. deprioritizing routine efforts). In the end, this decoupling exposed the polio programme to more risk as pockets of low routine coverage and the inability of the RI system to support IPV introduction have contributed to the emergence of cVDPV, including recent cases detected in Afghanistan, the DRC, Ethiopia, Indonesia and Nigeria. Other contextual factors (e.g. conflict and insecurity, declining political commitment, and community mistrust) further influenced these dynamics leading to policy decisions that, in responding to implementation challenges, often exacerbated distortion and limited sustainability. Recent GPEI documents suggest that persistent vaccine resistance as well as expanding cVPDV outbreaks, exacerbated by COVID-19 suspensions of polio campaigns, are finally changing attitudes in favour of integration even among the most resistant polio actors (GPEI, 2020a; IMB, 2020). Practical changes on the ground, however, remain limited and uneven as implementers grapple with how to move towards integration at this stage of the initiative and in complex settings.

Naturally, the extent and nature of these dynamics varied between countries and at different levels within each country. Health systems are rarely fully ‘weak’ or fully ‘strong’, and effects (both positive and negative) were sometimes more acute in a given arena or locality than others. A systems-thinking approach highlights how fundamental system characteristics, such as system feedback loops and links between sub-systems, can regulate the impact of public health interventions on the health system (Adam and de Savigny, 2012; Peters, 2014). In this case, the GPEI has become increasingly effective at overcoming environmental barriers to implementation and extending coverage to hard-to-reach communities, such as through spatial mapping for household enumeration, emphasizing the level of effort and resources required to reach everyone with essential health services. These adaptations have been critical for the success of the polio programme and have increased capacity for service provision within the health system in the short term. Over the long term, however, what may appear to be a reinforcing cycle can in fact be a vicious one if these implementation strategies are not consciously adopted by other programmes and sub-systems and if the gravitational pull towards polio objectives draws too many resources away from other health system objectives.

### DDD framework for programmatic assessment: challenges and adaptations

Although both the design and domains of the DDD framework were applicable to our analysis, we made amendments as the study progressed. Given the specific, vertical nature of the polio programme, our additional focus on integration and parallelism was critical for unpacking the health system dynamics at play and for understanding why short-term developments may not succeed in creating long-term systems change. Future applications of the DDD should consider incorporating these domains to make explicit the impact of delivery and governance mechanisms on health system change, especially for large, disease-specific initiatives. During analysis, we also (1) found it was difficult to disentangle actors and functions as distinct domains and (2) felt the need for a more nuanced middle ground between development and distortion that recognized that not all changes precipitated by an external force are entirely beneficial or harmful. Future research that utilizes other systems thinking tools (e.g. causal loop and causal pathway diagrams Lewis *et al.*, 2018) may be helpful for addressing these limitations and assessing the effects of these dynamics (Alonge *et al.*, 2017) as they are: multi-layered, interdependent and evolving over time. The GPEI is a unique example of a potentially disruptive intervention, but we feel the DDD framework presents considerable analytical promise for understanding how vertical health programmes interact with the health systems in which they are implemented. We believe our paper also answers the call for integrating more social science perspectives and research into analyses of global health programmes and their interaction with the health system (Bardosh, 2014) and contributes to understanding the ‘inherently political processes’ and power dynamics that national and global/multilateral actors navigate to achieve their health goals (Kickbusch, 2015).

There are several limitations to our work. First, the respondents for this analysis only represent a small proportion of the thousands of people that have worked on polio eradication from the front lines to global strategic positions. However, by using a purposive approach to our respondent selection, we believe we have included perspectives that cover the history of the programme, critical activities around implementation, and views from multiple levels of implementation across contexts. Second, although we reinforced the historical nature of our inquiry during interviews, respondents may experience a recency bias in their responses with impressions of countries’ current challenges at the forefront of their minds. Third, country case studies represent the tail end of eradication efforts, so we have limited first-hand information from those working in contexts that achieved elimination early on, e.g. in the Americas region (Pan-American Health Organization, 1995). Finally, due to the nature of our data collection, we were not able to analyse the cost domain of the DDD framework.

### Strategies for future disease control efforts

Assessment of the GPEI’s impact offers considerations for future disease control, elimination or eradication efforts that could mitigate distortion and maximize development of health systems. In recognition of the inherent power differentials between global and national actors, we direct these considerations particularly to global actors in their role as supra-country institutions that drive financing and implementation of international disease control. First, new eradication efforts should be approached carefully by engaging directly with questions about (1) whether eradication is the right goal, (2) whether eradication (or even elimination) is an equal priority for country and global stakeholders, (3) which criteria are being used to justify a new eradication effort and who is setting those criteria and (4) what the trade-offs are between elimination and eradication. Any future eradication conversations need to give equal weight to sociopolitical feasibility as they do to technical feasibility, specifically evaluating the epidemiological benefit against the geopolitical, social and ethical trade-offs of pushing a global effort that may not have buy-in from every country or the political stability for a successful eradication programme (Aylward *et al.*, 2003; Taylor, 2009; Yekutiel, 1980). Furthermore, careful attention should be paid to any endgame rhetoric that is focused on targets and deadlines, which can lead to coercion (White, 2020).

Second, transition planning is essential at the outset of any global health initiative, especially disease elimination and eradication programmes, and there should be agreement from all stakeholders in advance on this point. Planning with the end in mind will change the choices a programme makes around its design and implementation, including issues about integration, financing, stakeholder engagement and risk tolerance and will lay a better groundwork for ensuring sustainability of programmatic gains achieved. The benefits and investments necessary for transition planning have been raised before (Bennett *et al.*, 2015; Sgaier *et al.*, 2013).

Third, future programmes should leverage and integrate their investments into other local programmes, such as including other health workers in capacity building efforts, overhauling payment systems for all outreach workers and establishing programme activities with existing staff cadres. New programmes should not become the backbone of the system nor should they offer incentives or payments that exceed local system limits; otherwise, the whole system could collapse once the programme ends.

Last, equity and inclusion should be core tenets from the start. The polio programme’s shift towards an approach that truly focuses on ‘reaching every child’ was forced, in part, by missed targets and deadlines. Future programmes should begin with the last 1% as their target as they represent the most underserved, excluded and marginalized populations that a health system can serve. The polio programme’s efforts also indicate that designing with equity in mind is more likely to result in programme success and support health system developments.

## Conclusion

The 30+-year effort to eradicate polio worldwide has made enormous contributions to public health and averted poor outcomes for many; however, polio eradication has had a mixed track record when it comes to strengthening local health systems. While it has built capacity and structures where there were none, the optimistic belief in rapidly achieving eradication goals led to the short-sighted establishment of parallel systems that drew attention away from RI in pursuit of short-term goals. As eradication targets have been missed over time, sunk costs and the global-level pursuit of achievement have made course correction difficult. While the GPEI has become an incredibly large and influential force in global health pursuing a commendable goal, its attentions come at a cost that future programmes need to actively avoid.

## Supplementary Material

czab044_Supp

## Data Availability

The key informant data underlying this article cannot be shared publicly to protect the privacy of individuals that participated in the study. Key informant data may be shared on reasonable request to the corresponding author. Documentary data have been cited throughout the article.

## Endnotes

1. According to the Co-Chairs’ Summary of the 17^th^ High-level Meeting of the Global Polio Partners Group (September 2020), the Transition IMB has been reconstituted and will meet in the fall of 2020, but no records of meetings or reports were found as of mid-January 2021.

## Acronyms

BMGF - The Bill and Melinda Gates Foundation

CDC - U.S. Centers for Disease Control and Prevention

CSO - civil society organization

cVDPV - circulating vaccine-derived poliovirus

DDD - Develop–Distort Dilemma

DRC - Democratic Republic of Congo

EPI - Expanded Programme on Immunization

GPEI - Global Polio Eradication Initiative

HiT - Health Systems in Transition

IPV - inactivated poliovirus

NGO - non-governmental organizations

OPV - oral poliovirus

RI - routine Immunization

SIAs - supplementary immunization activities

TIMB - Transition Independent Monitoring Board

UNICEF - United Nations Children’ Fund

WHO - World Health Organization
