# Supplementary material for: The effects of polio eradication efforts on health systems: a cross-country analysis using the Develop–Distort Dilemma
Source: Health Policy Plan. 2021 Apr 21;36(5):707–19. doi: 10.1093/heapol/czab044 (PMC8173659; doi:10.1093/heapol/czab044)
Supplement: czab044_Supp [file czab044_supp.zip › GPEI DDD Paper_Suppl Files_2020 03 03.docx]

**The Effects of Polio Eradication Efforts on Health Systems: An Cross-Country Analysis using the Develop-Distort Dilemma – Supplementary Files**

***Polio program characteristics, by study country***

| Indicator^1, 2^ | Afghanistan | Bangladesh | Democratic Republic of Congo | Ethiopia | India | Indonesia | Nigeria |
| --- | --- | --- | --- | --- | --- | --- | --- |
| Country status | Endemic | Polio-free (2014) | Outbreak | At-risk* | Polio-free (2014) | Polio-free (2014)* | Endemic |
| Risk factors | - Low immunization coverage - Poor sanitation - Population movement internally and internationally - Poverty and poor social determinants of health (SDOH) - Insecurity and hard-to-reach - Lack of trust in polio program, vaccine refusal | - Risk of polio importation - Poor sanitation - Population movement internationally | - Low immunization coverage - Cases of cVDPV - Population movement internally and internationally - Poor sanitation - Poverty and poor social determinants of health - Conflict - Cases of cVDPV | - Low immunization coverage - Population movement internally and internationally - Risk of polio importation - Cases of cVDPV - Poor sanitation - Poverty and poor social determinants of health | - Lack of access to clean water plus poor sanitation - Mistrust in vaccination efforts | - Risk of polio importation - Inconsistent availability of IPV - Cases of cVDPV - Low coverage in pocket areas | - Low vaccination rates due to refusal or inaccessibility - Poverty and poor SDOH - Conflict/Insecurity and hard-to-reach - Lack of trust in polio program, vaccine refusal - Lack of access to clean water plus poor sanitation - Risk of polio importation - Population movement internally and internationally - Cases of cVDPV |
| Polio added to vaccination schedule | 1978 | 1979 | 1988 | 1980 | 1978 | 1981 | 1979 |
| IPV introduction | 2016 | 2015, fractional IPV from 2017 | 2015 | 2015 | 2016 | 2016 | 2014 (campaigns only); 2015 (RI) |
| Overall coordination of polio eradication activities | - National Polio Eradication Steering Committee - Presidential Focal Point for Polio Eradication | - Interagency Coordination Committee on Immunization, chaired by Ministry of Health and Family Welfare | - Strategic Interagency Coordinating Committee | - Maternal, Child Health and Nutrition Directorate | - Ministry of Health and Family Welfare | - Ministry of Health (Directorate General of Disease Control, Directorate General of Public Health, Directorate General of Pharmaceutical and Medical Devices) - Indonesia Technical Advisory Group on Immunization (ITAGI) - National Certification Committee for Polio Eradication (NCCPE) - Expert Review Committee (ERC) - National Authority for Containment (NAC) | - Presidential Task Force for Polio Eradication and Routine Immunization - National Emergency Operations Centre |
| Current polio program objectives | - Interrupt circulation of indigenous poliovirus - Increase population immunity - Respond to WPV importation - Maintain surveillance | - Maintain polio-free status | - Support delivery of vaccine - Strengthen supply chain - Strengthen surveillance system - Improve communication and stakeholder engagement | - Achieve polio-free status by 2018 | - Increase full immunization coverage to 90% by 2018 | - Detect and interrupt polio transmission and circulation - Enhance and strengthen immunization program - Polio virus containment - Use polio structure for other programs (legacy planning) | - Achieve and sustain polio free (WPV, cVDPV) status - Achieve and sustain surveillance performance indicators in all LGAs - Achieve 50% reduction in number of unimmunized children in VVHR LGAs - Transition Plan completed and approved by June 2018 - Increase reach of inaccessible areas in Borno |
| Current program-specific activities | - Improve routine immunization coverage - Supplementary Immunization Activities - AFP surveillance - Targeted mop-up campaigns | - National immunization days - AFP surveillance - Lab testing through National Polio Laboratory | - Reach Every District - Improve program management - Strengthen logistics - Improve monitoring and data quality - Strengthen communication of EPI - Integrate immunization with other MCH interventions - Strengthen surveillance - Build human and institutional capacity - Improve vaccine safety and regulation | - Strengthen routine immunization and expand coverage - SIAs and mass campaigns for additional doses - WPV surveillance by investigating AFP cases - Intensive, targeted mop-up campaigns | Focus on areas of low immunization coverage, incl. hard-to-reach areas, vacant health centers, areas with recent outbreaks, areas of vaccine hesitancy. | - Routine immunization - AFP surveillance - Supplementary Immunization Activities (SIA) eg. NIDs - Outbreak preparedness - Environmental surveillance - Laboratory containment | - Sustain resilience - Enhance SIA quality in prioritized vulnerable areas - Increase access to vaccination in security challenged areas and IDPs - Robust outbreak response in all states - Routine immunization in polio high risk LGAs - Surveillance - Cross-border collaboration - Improve quality assurance of all polio data   Polio transition planning |
| External support | Extensive; Gov’t only covers 5% of immunization program | GPEI ending n 2019  Gavi ending in 2022 | Substantial financing from external sources | Substantial financing from external sources, mainly GPEI which is ramping down. Resource mobilization includes engaging other donors to support program. | External support from Gavi, GPEI and others but most from gov’t of India | Funded by Government of Indonesia and Gavi support (2004-2017) | External support from WHO, UNICEF, Rotary, CDC, and Gavi |
| * Indonesia and Ethiopia were classified as "polio-free" and "at-risk" at the initiation of research, however, since then both countries have confirmed cases of circulating vaccine-derived poliovirus (cVDPV). Indonesia confirmed one case in Papua province in November 2018; Ethiopia has confirmed two cases to date in 2019, linked to an ongoing outbreak in Somalia.  ^1^All data from country program summaries and HiT tools unless otherwise noted  ^2^World Bank Data  ^3^WHO Statistics | | | | | | | |
